# Comparison of continuous temperature measurement methods in the intensive care unit: standard bladder catheter measurements versus non-invasive transcutaneous sensors

**DOI:** 10.1007/s10877-024-01199-2

**Published:** 2024-07-27

**Authors:** Ulrike Elisabeth Ehlers, Jens Ulmer, Mirja Keller, Carsten Klein, Urs Pietsch

**Affiliations:** 1 Division of Perioperative Intensive Care Medicine, Cantonal Hospital St. Gallen, Rorschacher Str. 95, St. Gallen, 9007 Switzerland; 2 Institute for Microtechnology and Photonics, Eastern Switzerland University of Applied Science, Werdenbergstr. 4, Buchs, 9471 Switzerland; 3 Swiss Air-Ambulance Rega, Bimenzälterstr. 87, Zürich-Flughafen, 8058 Switzerland

## Abstract

The purpose of this study was to compare a wearable system for body core temperature measurement versus bladder and tympanic thermometers in an intensive care setting. The question was, if continuous non-invasive sensors in the intensive care unit represent an alternative to current standard methods of invasive continuous bladder temperature measurement methods?

Between May and September 2023, a comparative investigation involving 112 patients was conducted in a 20-bed surgical intensive care unit to assess various temperature probes, including those placed in the tympanic tube, bladder, and skin. To achieve this, a wireless non-invasive sensor system provided by greenTEG AG, Switzerland, was affixed to different body locations (clavicular and lateral chest) of each catheterized patient (equipped with a temperature probe) admitted to the intensive care unit. Furthermore, tympanic temperatures were recorded at specified intervals. The measurement duration ranged from a minimum of six hours to a maximum of six days, resulting in the analysis of a total of 355 simultaneous temperature measurements.

In this study, a wearable temperature measurement system attached to two different body sites revealed a consistent negative bias compared to bladder temperature. In addition, the measurements were particularly influenced by body constitution. The tested system in all patients showed a mean absolute error (MAE) of 0.45 °C for the lateral chest and 0.50 °C for the clavicular position. Tympanic measurements had a mean absolute error of 0.35 °C. In patients with body mass index (BMI) ≥ 25 the MAE increased to 0.5 °C for the lateral chest and 0.56 °C for the clavicular position. In contrast, the tympanic measurement had a reduced MAE of 0.32 °C, which is well below this threshold when compared to bladder measurements.

In conclusion the investigated system did not meet the clinically relevant acceptance criteria and showed low precision in correctly identifying fever episodes compared to invasive temperature probes, however its main advantage lies in its continuity and non-invasiveness. This makes it a potential alternative to intermittent tympanic measurement devices. In this study we were able to show, that in at least one subset of patients, the non-invasive and continuous device demonstrated a precision comparable to tympanic measurements.

The accuracy of all non-invasive methods was lower than in previous studies, suggesting that the use of bladder temperature as reference and user related variations may have introduced additional errors.

**Supplementary Information:**

The online version contains supplementary material available at 10.1007/s10877-024-01199-2.

## Introduction

Temperature management is a common practice in intensive care units (ICUs) and requires precise temperature monitoring systems. Fluctuations in body temperature provide valuable insight into a patient’s health status and play a central role in the clinical decision-making process. The average human body core temperature is 36.6 °C (normothermia) within a range of 35.7 °C to 37.3 °C [1, 2]. Lower body, or hypothermic temperatures are mainly associated with external factors, such as cooling due to low environmental temperatures. Conversely, temperatures exceeding 38 °C are classified as hyperthermia or fever [3].

Individuals with a higher BMI generally display slightly elevated body temperatures, whereas aging is associated with a notable decrease in body temperature, often reaching approximately 35 °C. Despite these trends, establishing a specific target value for normothermic body temperature remains challenging, given the influence of environmental conditions, exercise, and illness on temperature regulation [2–4].

Moreover, variations in temperature readings can arise from different measurement sites. Tympanic probes, for instance, result in values that closely align with body core temperature, comparable to measurements taken within the bladder or pulmonary artery. The observed deviation typically falls within the range of 0.2 °C to 1 °C and are influenced by operators or presence of cerumen [5–7].

Changes in body temperature exerts optimized treatment through tailored individual temperature management. Challenges may arise, especially concerning compromised coagulation, when managing patients with temperatures below 35 °C [8–10]. Conversely, temperatures above 39.5 °C can occasionally lead to cell damage and organ dysfunctions [11]. Yet, there are instances where the advantages of non-normothermic states are favorable, such as after resuscitation or in cases of cerebral damage. In these situations, brief hypothermia is applied to reduce energy requirements [12].

In intensive care unit setting, more than 25% of patients have elevated temperatures during their stay, mostly due to underlying infectious diseases or drug-related illnesses. Tight temperature control is essential for effective patient management and the detection of possible infections. Therefore, fast and accurate temperature measurements are common practice in intensive care units using tympanic temperature probes [3, 5, 13]. The intermittency of this technology avoids continuous data recording and temperature change detection reducing the effectiveness of patient management [7]. For continuous temperature monitoring, bladder temperature probes and esophageal catheters, have been established [6]. However, despite their effectiveness in providing accurate and continuous temperature data, these invasive approaches come with certain drawbacks [14].

One significant drawback of invasive temperature monitoring techniques is the potential discomfort they can induce in patients. The insertion of probes or catheters can be invasive, painful, and disruptive to patients sometimes leading to serious injuries [15]. Moreover, these invasive methods carry a potential risk of infection, which may require regular changes of catheters increasing not only the cost of such systems but also requires additional medical treatment.

In response to these challenges, there has been a growing interest in the development and adoption of non-invasive continuous temperature monitoring devices. Moreover, it will allow the continuous monitoring of non-catheterized patients giving advantages over intermittent tympanic measurement systems.

In this study, a new non-invasive body core temperature sensor was assessed alongside an intermittent tympanic temperature probe within the surgical intensive care unit of the Cantonal Hospital of St. Gallen, Switzerland. The two measurement methods were compared with a continuous invasive standard temperature sensor as reference.

## Methods

This single-center study at a 20-bed intensive care unit at the Swiss Cantonal Hospital of St. Gallen was approved by the cantonal ethics commission of St. Gallen (Req-2023-00304) and carried out from May 1, 2023 to September 30, 2023. The study was conducted in line with the principles of the Declaration of Helsinki and all data where anonymized. The aim of the study was to compare a novel non-invasive continuous temperature measurement method with the standard methods (continuous bladder catheter and intermittent tympanic measurement method). Patients from interdisciplinary surgical clients were treated (neurosurgical, visceral surgery, thoracic surgery, urology, gynecology, ear nose throat).

All patients aged 18 years and over who were hospitalized in the surgical intensive care unit and had a bladder catheter inserted were included. Exclusion criteria primarily related to the intolerance or impossibility of attaching the temperature adhesive sensors (in wounds, any allergies, existing inflammatory skin changes). Furthermore, patients who were externally cooled or warmed for therapeutic reasons and patients who did not have a bladder catheter inserted were excluded.

The intensive care unit’s study coordination managed patient screening, inclusion and exclusion, as well as the installation of temperature sensors. The temperature measurement and documentation were carried out by the responsible nursing specialist for each patient.

**Fig. 1:**
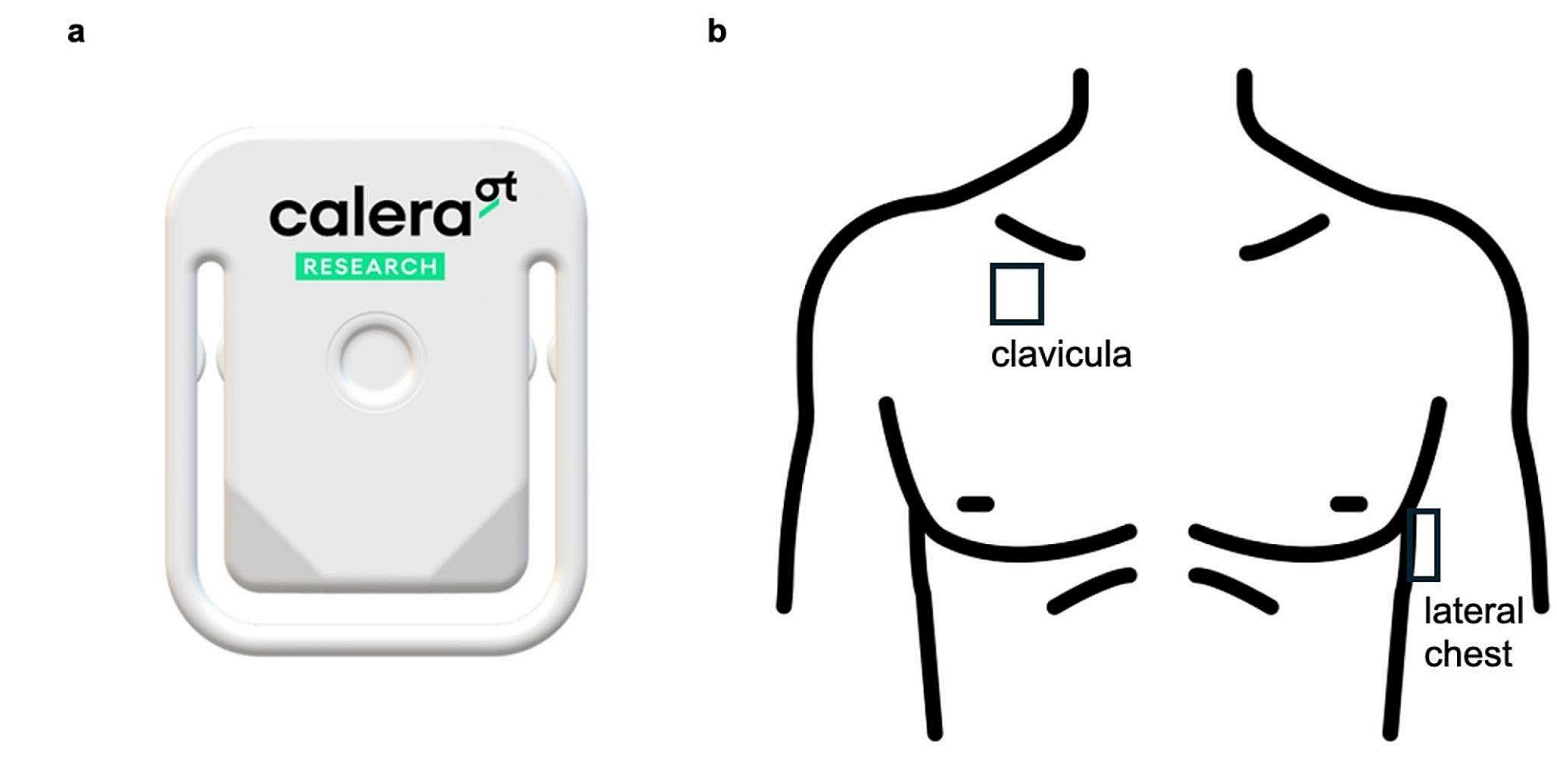
**a ** Non-invasive temperature sensor which is attached by adhesive tape on the upper torso. **b** Positions of the attached senor and it’s definitions

For all subjects, body temperature was assessed intermittently through tympanic measurements, continuously using bladder catheters and wirelessly using the skin temperature sensors. Continuous data recording from the bladder catheter began immediately upon patient admission and continued until transfer from the intensive care unit. The adhesive skin sensors were applied upon patient entry, with one positioned approximately 1 cm below the mid-collarbone on the right side (clavicular) and another about 15 cm below the armpit on the left side at heart level on the upper body (lateral chest, see also Fig. 1).

Initial measurements were taken after a 15-minute equilibration period. The devices were expected to provide temperature readings for a minimum of 6 h and a maximum of 6 days. Tympanic temperature measurements were conducted at entry and discharge, as well as at least once every 8 h during the stay.

The tympanic temperature probe used in the study was a WelchAllyn Braun ThermoScan Pro6000 (Hill-Rom SA, Switzerland) using a replaceable probe cover to control thermometer placement during the measurement. The accuracy is specified with ± 0.2 °C in the range of 35 °C to 42 °C.

The bladder temperature was recorded with a sterile foley urinary bladder catheter from Envisen (Anandic Medical Systems, Switzerland). The accuracy is specified with ± 0.1 °C in the range of 25 °C and 45 °C.

For non-invasive temperature measurement, we employed a wireless sensor system. The sensors were supplied by greenTEG AG (Rümlang, Switzerland). The device predicts body core temperature continuously using built-in heat flux and skin temperature sensors. Calculated temperature values are transmitted to a mobile device using Bluetooth and ANT communication protocols. In this study, the CALERAresearch model 2022-Q4 with firmware FeverFlux 0.8.1 has been used.

### Data analysis

The data collection was carried out by the study management and coordination and was anonymized.

The statistical analysis was carried out after coding the data collection. Data structuring, statistical calculations and data plotting was done in python (v.3.9, Scotts Valley, CA: CreateSpace, USA).

Bland-Altman statistics were used to compare the difference between multiple measurement sites and the reference measurement (bladder temperature) [16]. Due to the intermittent nature of tympanic temperature measurement, only those time points in the data set were chosen where all 4 measurements showed a valid temperature value. Temperature validity was defined as a positive temperature value in all 4 devices larger than 36.0 °C as the non-invasive temperature sensor was not capable to calculate body core temperatures correctly below this value. Due to patient manipulation, catheter change, or other procedures, data cleaning was necessary. Tympanic measurement values below 34 °C and above 42 °C were also excluded from the analysis as these were considered faulty measurements. The quadruplet of data consisted of the following temperature measurement sites:

- Non-invasive Temperature measurement with CALERAresearch in the lateral chest position: T_cl_.
- Non-invasive Temperature measurement with CALERAresearch in the clavicular position: T_cc_.
- Non-invasive Temperature measurement with Tympanic thermometer: T_t_.
- Invasive Temperature measurement with Bladder thermometer: T_b_.

In addition to Bland-Altman analysis, the concordance correlation coefficient according to Lin was calculated to evaluate the agreement between pairs of observations visualizing systemic bias and random error [17]. A sensitivity and specificity analysis were used to define sensor performance to detect febrile conditions (Tb ≥ 38 °C). To test the significant difference between the different sensor positions or different patient sub-populations a z-statistical analysis by calculating the diagnostic odds ratio and the standard error of the log odds ration with its 95% confidence intervals were calculated [18].

Additionally, we computed the percentage of differences falling within a predefined threshold of ± 0.5 °C compared to T_b_.

Using error grid analysis, we estimated the percentage of false positive and false negative fever episodes based on a bladder temperature (T_b_) threshold of ≥ 38 °C. These inaccuracies could potentially lead to misdiagnoses during the clinical decision-making process. Following Bräuer et al. recommendations the different zones were defined as [19]:

- Zone A represents the clinically insignificant area where differences between the predictive values (T_cl_, T_cc_, T_t_) and the reference value (T_b_) would lead to the same clinical decision, regardless of whether the bias is below or above 0.5 °C, or whether the febrile threshold of ≥ 38 °C has been reached.
- Zone B identifies the area of false positive febrile event detection, where the predictive values exceed the fever threshold of 38 °C, but the reference values are at least 0.5 °C below this threshold. In this zone, the clinical decision could result in unnecessary medical treatment.
- Zone C describes biases in the predictive values (T_cl_, T_cc_, T_t_) greater than 0.5 °C that could lead to incorrect clinical decisions and potentially harm the patient. For example, if T_b_ records 38.5 °C and Tc shows 37.6 °C, the patient might not receive fever-lowering therapies, even though they are clinically indicated (false negative event).

### Results

### Demographics

Between May 2023 and September 2023, we enrolled 112 patients at the intensive care unit in SG. Patient characteristics are summarized in Table 1.

**Table 1:** Clinical characteristics of study patients (*N* = 112). SD: standard deviation, BMI: body mass index, NOR: Norepinephrin

Clinical Characteristic	
Age ± SD	63.3 ± 15.1 years
Weight ± SD	76.0 ± 16.2 kg
Height ± SD	170.4 ± 9.7 cm
BMI ± SD	26.1 ± 4.6 kg/m^2^
Sex, male/female	64/48
Total valid datapoints	355
Fever episodes	74
BMI, > 25/ ≤ 25	60/52
No. with NOR	48

In total 365 quadruple temperature measurements were defined as valid. A total of 605 datapoints were excluded from the analysis due to data cleaning procedures, incorrect tympanic reading (< 35 °C, > 42 °C) or missing datapoints from either bladder or tympanic temperature sensors. Main reason was a catheter change or flushing procedure, which lead to false reading form the bladder temperature probe. In addition, 10 quadruplet data points were excluded due to hypothermic (< 36 °C) temperature reading from the bladder temperature sensor. The study was carried out without any complications. No adverse events occurred. The adhesive patches used to attach the sensors to the skin were well tolerated. There was no evidence of allergic reactions or skin irritations.

### Bland-Altman and bias analysis

**Fig. 2:**
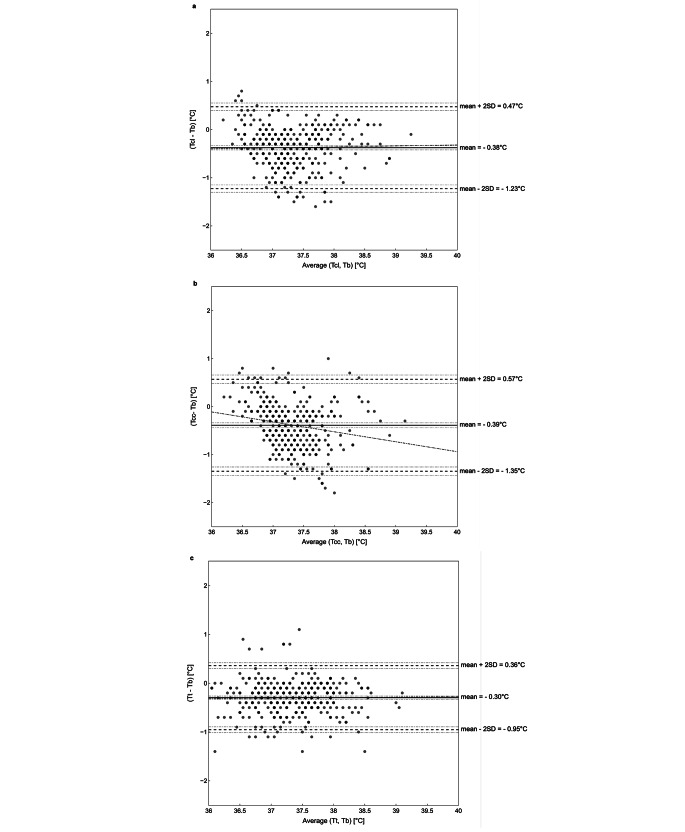
Bland-Altman Plots for temperature probes in lateral chest (**a**), clavicula (**b**) and tympanic (**c**) positions compared to bladder temperatures in all patients excluding hypothermic events. Displayed are the mean values and the 95% upper and lower limits of agreement as dashed lines. In addition, the CI of each parameter and the proportional bias are also indicated as dashed-dot lines. Shading of datapoints resembles the amount of datapoint at this temperature. Detailed information’s are summarized in Table S1 in the supplementary information

To compare the accuracy of the different temperature sensors a Bland-Altman analysis was performed. Across the entire patient cohort, the sensors on the lateral chest position exhibited an average bias of -0.38 °C compared to T_b_, with 95% limits of agreement ranging from − 1.23 °C to 0.47 °C. T_cc_ had a mean bias of -0.39 °C, with 95% limits of agreement within − 1.35 °C and 0.57 °C and T_t_ a mean bias of -0.30 °C, with 95% limits of agreement from − 0.95 °C to 0.36 °C (Fig. 2). For the detailed sub-group analysis please refer to Fig.S1 and Fig.S2 in the supplementary information.

**Fig. 3:**
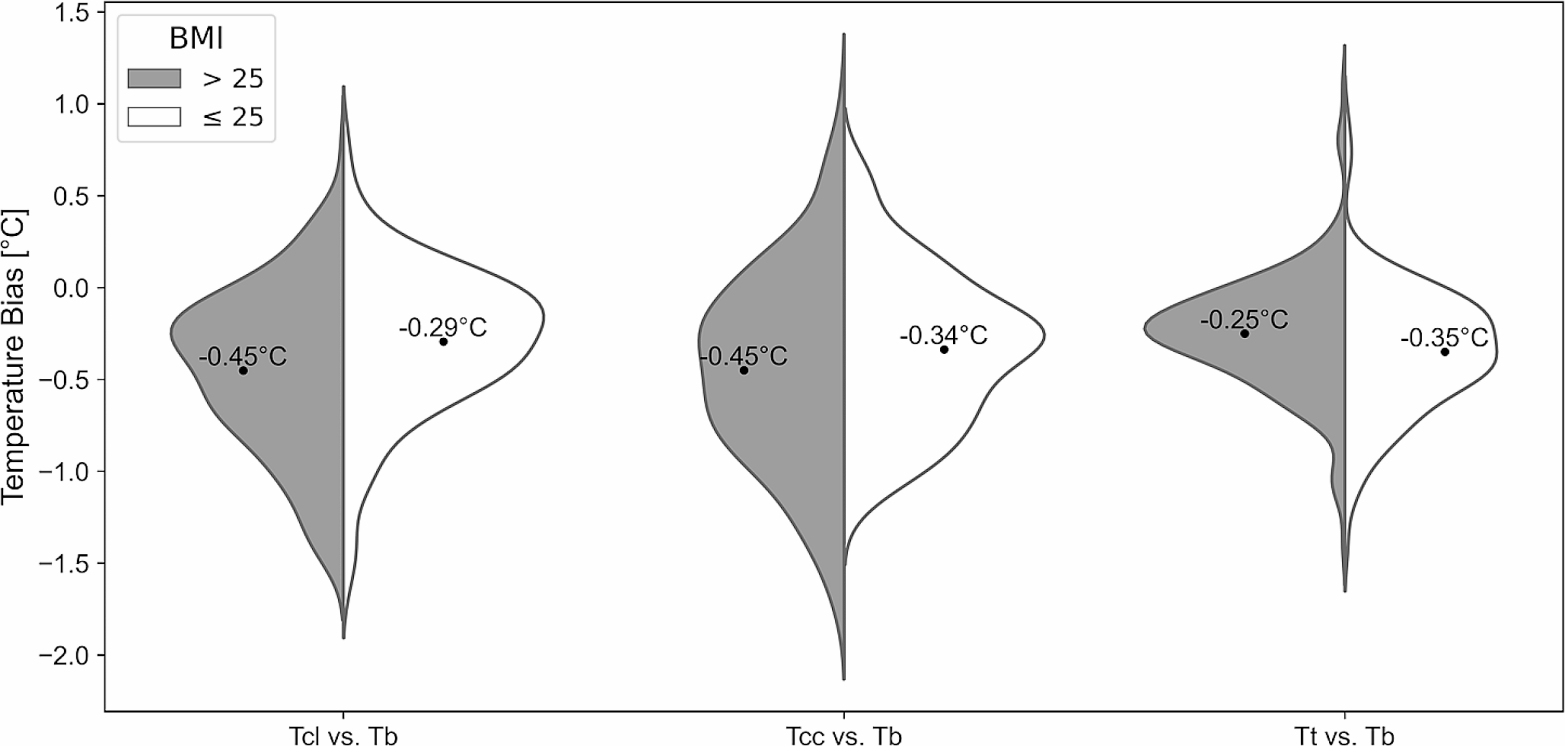
BMI Sub-group analysis of bias distribution for temperature probes in lateral chest (T_cl_), clavicula (T_cc_) and tympanic (T_t_) positions compared to bladder temperatures (T_b_). Mean values of bias are shown for each subset of data

The comparison of temperature measurements at various body sites reveals a distinct pattern of mean bias relative to the bladder temperature probe, particularly influenced by the patient’s BMI. Generally, the body core temperatures are underestimated more in patients with BMI > 25 compared to those with BMI ≤ 25 for both, lateral chest (-0.45 °C vs. -0.29 °C, *p* = 0.0007) and clavicular (-0.45 °C vs. -0.34 °C, *p* = 0.0274) positions (Fig. 3). The tympanic measurements, however, exhibit an opposite trend with a reduced bias in patients with a higher BMI (-0.25 °C vs. -0.35 °C, *p* = 0.008).

A detailed bias analysis revealed a proportional bias for sensors in the clavicula position, with an increased underestimation for higher core temperatures. The proportional bias was highest in the patient population with an BMI > 25 (slope: -0.39 °C; Fig.S1). For other values see Table S1.

### Numerical sensor performance

The MAE of the entire patient population was 0.45 °C for the lateral chest and 0.50 °C for the clavicular position. Tympanic measurements had a mean absolute error of 0.35 °C. The MAE changed when stratifying the data based on patients BMI. For patients with BMI > 25, the MAE was 0.50 °C for the lateral chest and 0.56 °C for the clavicular position, whereas the MAE for tympanic temperature decreased to 0.32°CFor patients with a BMI of 25 or less, the MAE was 0.37 °C for the lateral chest position, 0.43 °C for the clavicular site, and 0.40 °C for the tympanic site. To analyze the sensor performance, we calculated the proportion of errors within the rage of 0.5 °C. For the lateral chest position 64% and for the clavicula position 53% fall within the range of 0.5 °C, while 80% of tympanic measurements are within this range. The values from the sub-group analysis are listed in Table S1.

### Sensitivity and specificity for fever detection

**Fig. 4:**
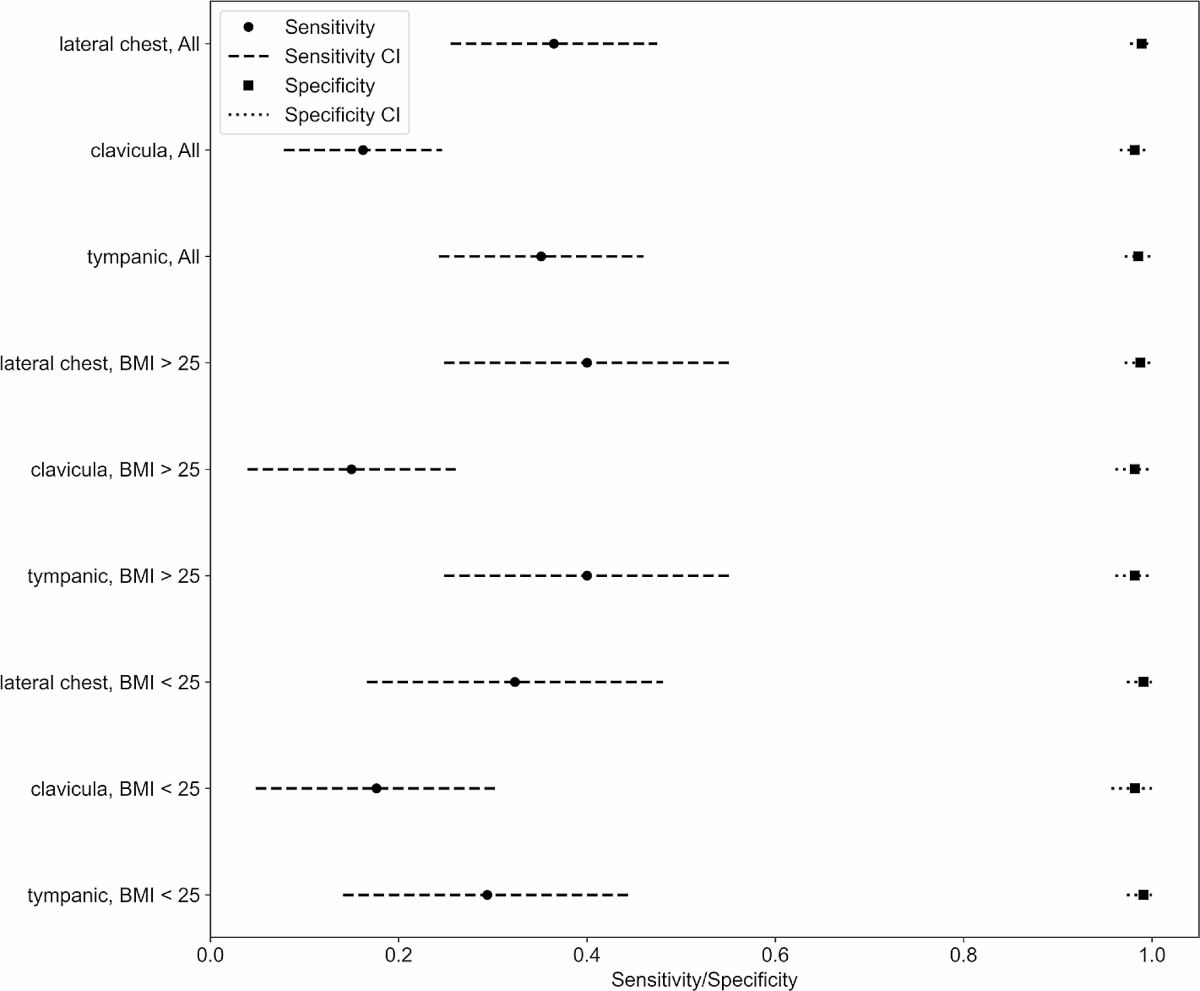
Sensitivity and specificity analysis illustrating the comparative performance of different sensor positions to detect fever where bladder temperature is ≥ 38 °C. The graph shows mean values of sensitivity and specificity along with 95% CI representing the diagnostic accuracy of each sensor position in identifying febrile conditions

A threshold for the sensitivity analysis in detecting febrile conditions was set to 38 °C. When using bladder temperature as a benchmark, the sensitivity for the wireless sensor in the lateral chest position was 36.5% in all patients (32.4% for BMI ≤ 25 and 40.0% for BMI > 25; Fig. 4). Sensitivity is most deficient at the clavicular site, with only 16.2% for all patients (17.6% for BMI ≤ 25 and 15.0% for BMI > 25). The tympanic probe’s sensitivity in detecting febrile conditions is limited to 35.1% in the entire patient cohort (29.4% BMI ≤ 25 and 40.0% for BMI > 25).

The diagnostic odds ratio calculations coupled with z-statistical analysis have not demonstrated any significant difference in sensitivity between lateral chest and clavicular sites. Similarly, the comparison of sensitivity and specificity among different BMI categories revealed no significant variations. The specificity remained notably high across all measurement methods and BMI groups, a factor likely influenced by the consistent negative bias, resulting in a false positive detection rate of fever below 1%.

### Lin’s concordance correlation coefficient

The association between the temperatures measured by the non-invasive sensor probes and the bladder temperatures is relatively weak. The correlation for the lateral chest position was 0.56 (0.63 BMI ≤ 25; 0.52 BMI > 25) and for the clavicular position 0.42 (0.58 BMI ≤ 25; 0.32 BMI > 25). The tympanic temperature probe had a modest correlation of 0.72 (0.67 BMI ≤ 25; 0.67 BMI > 25). Visualization of the correlation analysis can be found in the supplementary information (Fig. S3, Fig. S4 and Fig. S5).

### Diagnostic significance and grid analysis

**Fig. 5:**
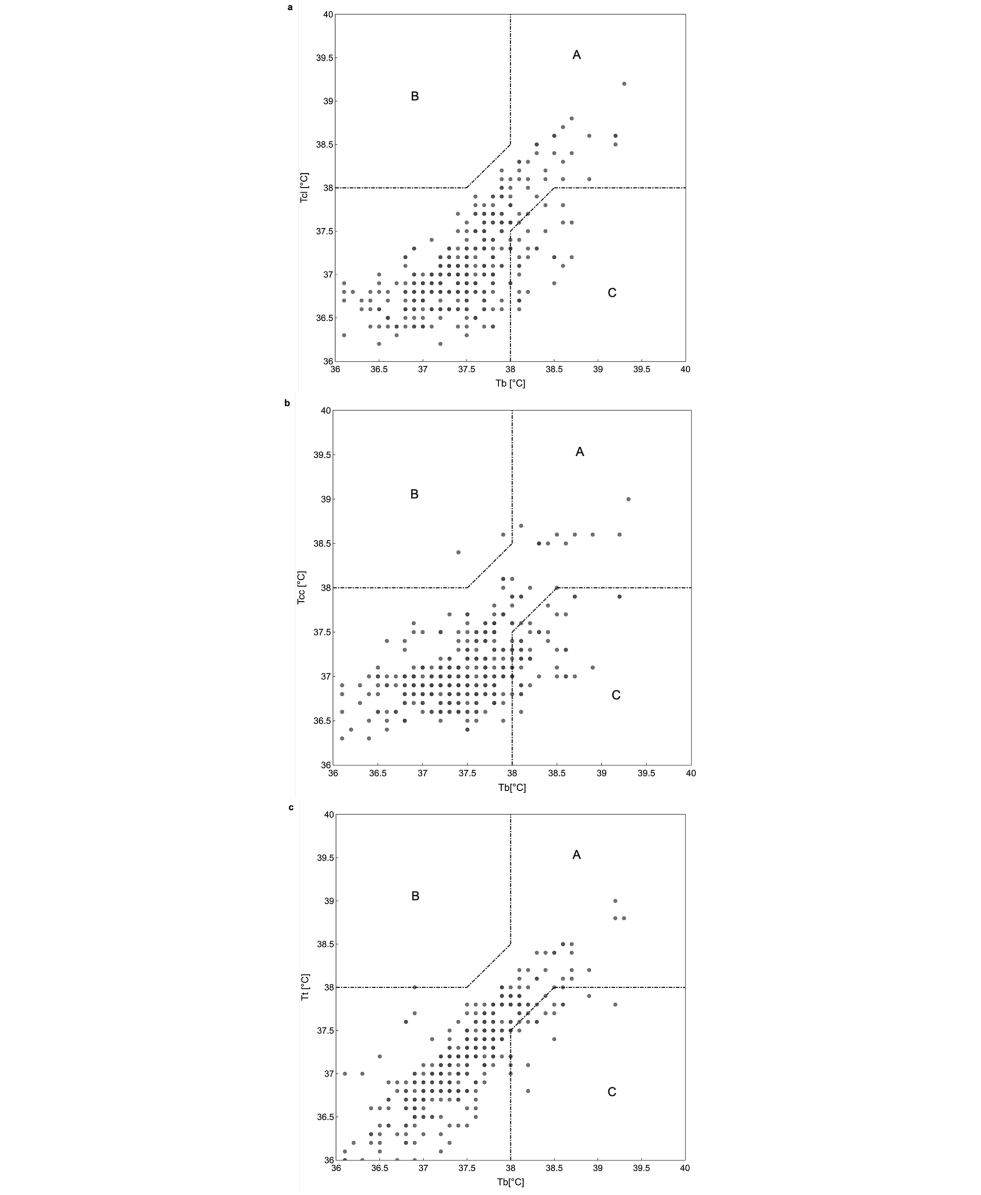
Error grid analysis for temperature probes in lateral chest (**a**), clavicula (**b**) and tympanic (**c**) position detecting febrile episodes in all patients. Zone A represents all episodes where measurements of predicting values (T_cl_, T_cc_, T_t_) and reference value (T_b_) did not indicate fever and would have resulted in the same clinical decision. Zone B represents all episodes where the predictive values (T_cl_, T_cc_, T_t_) indicated a fever event whereas the reference value did not (false positive). Zone C represents all episodes where the predictive values (T_cl_, T_cc_, T_t_) missed to indicate a fever event (false negative)

The grid analysis demonstrated that all three temperature measurement systems did not meet the clinical significance level of 95%. In at least 90.1% of cases, the clinical decisions based on lateral chest position measurements would have aligned with those made using bladder temperature measurements (Fig. 5; Fig. S6 and Fig. S7 for the patient sub-population analysis). For the clavicular position 84.2% of measurements would have led to the same clinical decision. Tympanic measurements also reached only a significance level of 93.2%. Further examination via a BMI subgroup analysis showed that the significant level of 95% could not be achieved, although tympanic measurements in overweight patients came close to 94.2%. Across the various patient BMI categories, no significant differences were noted.

The analysis also uncovered trends in clinically significant discrepancies. Specifically, in the lateral chest position 9.9% of measurements were false negative with clinical significance, leading to incorrect clinical decisions, missing febrile conditions. The clavicular temperature sensor would have led to a missed fever diagnosis in 15.2% of all cases. Tympanic measurements had a 5.7% incidence of missing fever, which could result in erroneous clinical decisions. The false positive rates for all temperature probes were below 1% where the sensors would have registered a febrile episode and leading to a wrong clinical decision.

### Influence of vasoactive drugs

To evaluate whether vasoactive drugs affected the non-invasive sensors, we categorized the patients based on their administration of norepinephrine. The analysis indicated that for patients receiving norepinephrine, the bias across the different sensor locations was slightly reduced by at least 0.1 °C (lateral chest: +0.13 °C (*p* = 0.04), clavicula: +0.16 °C (*p* = 0.02), tympanic: +0.1 °C (*p* = 0.06) indicating that with norepinephrine temperature underestimation is reduced.

## Discussion

Non-invasive body core temperature monitoring has been established for critically ill patients for over a decade and non-invasive probes are already in clinical use [20]. In this study, the CALERAresearch non-invasive and wireless temperature probe was analyzed by comparing it to continuous bladder temperature and intermittent tympanic probes across a cohort of 112 ICU patients. The CALERAresearch probe in the lateral chest position (T_cl_) exhibited a mean bias of -0.38 °C and revealed a large overall Limits of Agreement (LOA) of ± 0.85 °C that did not align with the criteria of ± 0.5 °C for clinically insignificant variations in temperature estimation [21–24]. Specifically, in overweight patients, discrepancies increased, with an overall LOA reaching ± 1.0 °C for the clavicular position (T_cc_). This finding highlights the significant impact of patient weight on temperature measurement accuracy, with normal-weight patients exhibiting lesser deviation from bladder temperature across all measurement sites compared to patients with a BMI over 25.

The accuracy and precision of the CALERAresearch probe were found to be position-dependent, with a notable bias in the clavicular position where it underestimated higher temperatures. This can be mainly attributed to the algorithm used to calculate the body core temperature, which is optimized for the lateral chest position. Furthermore, none of the positions estimated body core temperature accurately, with only 64% (lateral chest) and 53% (clavicular) of measurements falling within the range of ± 0.5 °C. The low accuracy can be mostly attributed to body mass index, as a subgroup analysis of normal-weight patients with a sensor placed in the lateral chest position showed a similar proportion of measurements within the 0.5 °C criteria compared to tympanic measurements (lateral chest: 73% vs. tympanic: 75%). However, accuracy remains limited when compared to other non-invasive temperature probes where the proportion of difference within 0.5 °C ranges between 84 and 94% [20, 25, 26].

For example probes based on zero heat-flux technology (SpotOn, 3 M Medical, St. Paul, Mn, USA) attached to the forehead estimate body core temperature with a negligible mean bias when compared to traditional bladder temperature methods [27]. Additionally, they demonstrate a 95% LOA within the acceptable range of ± 0.5 °C, affirming their reliability and use in ICU environments [25]. However, other technologies that measure skin temperature over the temporal artery (TAT-5000, Exergen Corp., Watertown, MN, USA; TA in Table 2) have failed to meet the critical threshold criteria of ± 0.5 °C and, thus, are not recommended for ICU settings (refer to data overview in Table 2) [19, 28–31].

**Table 2:** Mean Bias, number of measurements and 95% limit of agreement of different technologies to measure non-invasive body core temperature versus bladder temperature [28]

Reference	Technology	Mean Bias [°C]	*N*	(95% CI, LOA) [°C]
Own, apical	CALERAresearch	-0.38	355	(-1.23, 0.47)
Own, clavicula	CALERAresearch	-0.39	355	(-1.35, 0.52)
Schell-Chaple [27]	SpotOn	-0.07	748	(-0.54, 0.40)
Park [30]	SpotOn	0.07	679	(-0.51, 0.65)
Kimberger [28]	Tcore	-0.13	258	(-0.65, 0.40)
Stellfox [31]	TA	-0.14	736	(-1.70, 0.90)
Kimberger [29]	TA	0.07	280	(-1.48, 1.62)

In previous studies, the CALERAreseach device showed better agreement with a mean difference of 0.11 ± 0.34 °C (95%CI -0.55 to 0.77 °C) when compared to tympanic temperature estimations [32]. The comparison to tympanic measurements in all patients in the present study revealed a mean bias of -0.09 °C ± 0.97 °C (95%CI -1.05 to 0.89 °C) and − 0.08 °C ± 0.96 °C (95%CI -1.05 to 0.87 °C) for the lateral chest and clavicular positions, respectively.

Besides sensor performance, non-invasive temperature probes should also be able to correctly detect episodes of elevated body core temperatures, which was defined as T_b_ ≥ 38 °C. The accuracy in detecting fever episodes was 86% for the lateral chest position and 81% for the clavicular position for the entire patient population, which is still lower than previously reported by different technologies. However, the fever detection rate of the tympanic temperature probe was also lower than reported in other studies at 86% [33].

Due to the large negative bias of the investigated device, we also analyzed the clinical significance of missed fever episodes by an error grid analysis. Clinical significances were defined as those values which led to a non-diagnosed fever event indicated by a bladder temperature equal or above 38 °C. The false negative rate was up to 15.2% in the clavicular position, much higher compared to other studies, which showed percentages of measurements leading to incorrect clinical decisions between 0.6% and 2.2% [19, 25, 32]. For normal-weight patients, the difference in clinically significant incorrect temperature estimations was in the same range when comparing the lateral chest position with the tympanic measurements (lateral chest: 9.5%, tympanic: 6.8%), but again much higher than previously reported.

Another important factor, which had not been previously investigated, was the impact of vasoactive drugs during the use of heat flux-based temperature probes. Vasoactive medications, such as norepinephrine, are known to restrict blood circulation in the superficial skin, particularly in the body’s periphery [34]. In this study, there was a statistically significant change in mean bias for patients receiving norepinephrine. However, with 0.1 °C these differences are well below the threshold for clinical significance (± 0.5 °C). In addition, due to the limited number of fever episodes in this subset of patients, it was not possible to analyze the performance of detecting fever episodes in patients receiving norepinephrine.

### Limitations of the study

Non-invasive body core temperature estimation by heat-flux sensors is known to respond to temperature changes not as quickly as invasive probes. The different situations and medical treatments within a critical care unit might cause larger temperature variations within a shorter period. The dynamic response of the present sensors was not investigated in detail but might reflect a limitation within this study and explain some part of the negative bias of the CALERAresearch device.

The large discrepancies in the measurement results can be partly explained by the variability of the bladder temperature probe. It is known that bladder temperature measurements have very low bias from pulmonary artery measurements, ranging between 0.02 °C and 0.05 °C, but exhibit a LOA between ± 0.5 °C and ± 1.1 °C [35]. Additionally, the accuracy of bladder temperature probes is also affected by urinary outflow, which compromises the bladder temperature probe as an ideal standard for reliable temperature readings [36].

Additionally, it was shown that body composition influences temperature estimations. Different body types might affect how the device estimates temperature correctly, especially subcutaneous fat might impact precision and accuracy. Another limitation can be attributed to large body temperature fluctuations due to fluid management and large volume infusion to maintain volume status. This can temporarily affect body temperature, and maybe increase readout bias compared to the reference measurement.

## Electronic supplementary material

Below is the link to the electronic supplementary material.

Supplementary Material 1

## Data Availability

No datasets were generated or analysed during the current study.
