# Supplementary material for: Comparison of continuous temperature measurement methods in the intensive care unit: standard bladder catheter measurements versus non-invasive transcutaneous sensors
Source: J Clin Monit Comput. 2024 Jul 27;39(1):193–203. doi: 10.1007/s10877-024-01199-2 (PMC11821743; doi:10.1007/s10877-024-01199-2)
Supplement: Supplementary file 1 — Supplementary Material 1 [file 10877_2024_1199_MOESM1_ESM.docx]

Supplemetary informations:

**Table S1** Overview of statistics used in publication.

**Fig. S1** Bland Altman plots of patient sub-populations BMI ≤ 25

**Fig. S2** Bland Altman plots of patient sub-populations BMI > 25

**Fig. S3** Correlation plot of all patients

**Fig. S4** Correlation plot of patient sub-population BMI ≤ 25

**Fig. S5** Correlation plot of patient sub-population BMI > 25

**Fig. S6** Error Grid analysis of febrile detection of patient sub-populations BMI ≤ 25

**Fig. S7** Error Grid analysis of febrile detection of patient sub-populations BMI > 25

|  | BMI  Group | Mean Bias  (SD) [°C] | 95% LOA [°C] | MAE  [°C] | Error, 0.5°C [%] | Prop. Bias | LCCC (95% CI) | Sens [%] | Spec [%] | PPV [%] | NPV [%] | DOR, SE, (95% CI) | A  [%] | B  [%] | C  [%] |
| --- | --- | --- | --- | --- | --- | --- | --- | --- | --- | --- | --- | --- | --- | --- | --- |
| Lateral chest-Bladder | all | -0.38 (0.43) | -1.23, 0.47 | 0.45 | 64 | -0.05 | 0.56 (0.49-0.64) | 36.5 | 99.0 | 90.0 | 85.2 | 51.7, 0.63 (15.1-177.3) | 90.1 | 0 | 9.9 |
| Clavicular-Bladder | all | -0.39 (0.49) | -1.35, 0.57 | 0.50 | 53 | -0.27 | 0.42 (0.33-0.51) | 16.2 | 98.3 | 96.0 | 79.9 | 10.4, 0.66 (3.5-30.5) | 84.2 | 0.6 | 15.2 |
| Tympanic-Bladder | all | -0.30 (0.33) | -0.95, 0.36 | 0.35 | 80 | -0.04 | 0.72 (0.66-0.78) | 35.1 | 98.6 | 97.6 | 84.2 | 36.4, 0.56 (12.2-109.1) | 93.2 | 0.3 | 5.7 |
| Lateral chest-Bladder | ≤ 25 | -0.29 (0.40) | -1.07, 0.51 | 0.37 | 73 | -0.11 | 0.63 (0.52-0.75) | 32.4 | 99.1 | 91.6 | 82.6 | 52.1, 1.07 (6.4-423.9) | 90.5 | 0 | 9.5 |
| Clavicular-Bladder | ≤ 25 | -0.34 (0.41) | -1.15, 0.48 | 0.43 | 61 | -0.15 | 0.58 (0.46-0.70) | 17.6 | 98.2 | 75.0 | 79.4 | 11.6, 0.84 (2.2-60.5) | 83.8 | 0.7 | 15.5 |
| Tympanic-Bladder | ≤ 25 | -0.35 (0.34) | -1.04, 0.33 | 0.40 | 75 | -0.08 | 0.67 (0.57-0.77) | 29.4 | 99.1 | 90.9 | 82.0 | 45.4, 1.07 (5.5-371.8 | 91.9 | 0 | 6.8 |
| Lateral chest-Bladder | > 25 | -0.45 (0.44) | -1.31, 0.42 | 0.50 | 57 | -0.02 | 0.52 (0.42-0.62) | 40.0 | 98.8 | 88.9 | 87.0 | 53.6, 0.78 (11.6-248.2) | 89.9 | 0 | 10.1 |
| Clavicular-Bladder | > 25 | -0.45 (0.53) | -1.48, 0.60 | 0.56 | 48 | -0.39 | 0.32 (0.19-0.44) | 15.0 | 98.2 | 66.7 | 82.5 | 9.4, 0.73 (2.2-39.5) | 84.5 | 0.5 | 15.0 |
| Tympanic-Bladder | > 25 | -0.25 (0.32) | -0.88, 0.37 | 0.32 | 84 | -0.02 | 0.76 (0.69-0.84) | 40.0 | 98.2 | 84.2 | 87.0 | 35.6, 0.66 (9.6-131.2) | 94.2 | 0.5 | 3.9 |

**Table S1** Data overview of sub-group analysis for different temperature sensor positions compared to bladder temperature as reference. BMI: Body mass index; SD: Standard deviation; LOA: Limit of agreement; MAE: Mean absolute error; Error 0.5°C: Number of measurements within a 0.5°C threshold; Prop. Bias: Proportional bias from the corresponding bland-altman plot; LCCC: Lin’s concordance correlation coefficient; CI: Confidence interval; Sens: Sensitivity for detecting fever (T_b_ ≥ 38°C); Spec: Specificity for detecting fever (T_b_ ≥ 38°C); PPV: Positive predictive value for detecting fever (T_b_ ≥ 38°C); NPV: Negative predictive value for detecting fever (T_b_ ≥ 38°C); DOR: diagnostic odds ratio; SE: Standard error; A: Numbers of events in Zone A; B: Numbers of events in Zone B; C: Numbers of events in Zone C.

**Fig. S1** Bland-Altman Plots for temperature probes in lateral chest (**a**), clavicula (**b**) and tympanic (**c**) positions compared to bladder temperatures (T_b_) in patients with a BMI ≤ 25 excluding hypothermic events. Displayed are the mean values and the 95% upper and lower limits of agreement. In addition, the CI of each parameter are also indicated. Shading of datapoints resembles the amount of datapoint at this temperature. Detailed information’s are summarized in Table S1 in the supplementary information.

**Fig. S2** Bland-Altman Plots for temperature probes in lateral chest (**a**), clavicula (**b**) and tympanic (**c**) positions compared to bladder temperatures (T_b_) in patients with a BMI > 25 excluding hypothermic events. Displayed are the mean values and the 95% upper and lower limits of agreement. In addition, the CI of each parameter are also indicated. Shading of datapoints resembles the amount of datapoint at this temperature. Detailed information’s are summarized in Table S1 in the supplementary information.

**Fig. S3** Correlation analysis for temperature probes in lateral chest (**a**), clavicula (**b**) and tympanic (**c**) positions compared to bladder temperatures (T_b_) in all patients excluding hypothermic events. Displayed are the values of the corresponding sensor in relation to the reference temperature (T_b_). The line of equality is shown as dashed line. Shading of datapoints resembles the amount of datapoint at this temperature. Detailed information’s are summarized in Table S1 in the supplementary information.

**Fig. S4** Correlation analysis for temperature probes in lateral chest (**a**), clavicula (**b**) and tympanic (**c**) positions compared to bladder temperatures (T_b_) in patients with BMI ≤ 25 excluding hypothermic events. Displayed are the values of the corresponding sensor in relation to the reference temperature (T_b_). The line of equality is shown as dashed line. Shading of datapoints resembles the amount of datapoint at this temperature. Detailed information’s are summarized in Table S1 in the supplementary information.

**Fig. S5** Correlation analysis for temperature probes in lateral chest (**a**), clavicula (**b**) and tympanic (**c**) positions compared to bladder temperatures (T_b_) in patients with BMI > 25 excluding hypothermic events. Displayed are the values of the corresponding sensor in relation to the reference temperature (T_b_). The line of equality is shown as dashed line. Shading of datapoints resembles the amount of datapoint at this temperature. Detailed information’s are summarized in Table S1 in the supplementary information.

**Fig. S6** Error grid analysis for temperature probes in in lateral chest (**a**), clavicula (**b**) and tympanic (**c**) position detecting febrile episodes in patients with BMI ≤ 25 excluding hypothermic events. Zone A represents all episodes where measurements of predicting values (T_cl_, T_cc_, T_t_) and reference value (T_b_) did not indicate fever and would have resulted in the same clinical decision. Zone B represents all episodes where the predictive values (T_cl_, T_cc_, T_t_) indicated a fever event whereas the reference value did not (false positive). Zone C represents all episodes where the predictive values (T_cl_, T_cc_, T_t_) missed to indicate a fever event (false negative).

**Fig. S7** Error grid analysis for temperature probes in lateral chest (**a**), clavicula (**b**) and tympanic (**c**) position detecting febrile episodes in patients with BMI > 25 excluding hypothermic events. Zone A represents all episodes where measurements of predicting values (T_cl_, T_cc_, T_t_) and reference value (T_b_) did not indicate fever and would have resulted in the same clinical decision. Zone B represents all episodes where the predictive values (T_cl_, T_cc_, T_t_) indicated a fever event whereas the reference value did not (false positive). Zone C represents all episodes where the predictive values (T_cl_, T_cc_, T_t_) missed to indicate a fever event (false negative).
